# Serum total bilirubin and long-term prognosis of patients with new-onset non-ST elevation myocardial infarction: a cohort study

**DOI:** 10.1186/s12872-022-02607-8

**Published:** 2022-04-12

**Authors:** Yi Yang, Jun Wang, A Ji Gu Li Wai Si Ding, Yanan Xu, Haibing Jiang, Kezhong Ma, Tongjian Zhu

**Affiliations:** 1 Department of Cardiology, Institute of Cardiovascular Diseases, Xiangyang Central Hospital, Affiliated Hospital of Hubei University of Arts and Science, Xiangyang, Hubei China; 2 Department of Cardiology Fourth Ward, The Xinjiang Medical University Affiliated Hospital of Traditional Chinese Medicine, Urumqi, 830011 China; 3 Xinjiang Medical University, Urumqi, 830011 China; 4 Department of Cardiology, The People’s Hospital of Xuancheng City, Anhui, 242000 China; 5 Respiratory Medicine, The People’s Hospital of Xuancheng City, Anhui, 242000 China; 6 Department of Coronary Heart Disease, The Seventh Affiliated Hospital of Xinjiang Medical University, Urumqi, 830011 China

**Keywords:** Cohort study, Major adverse cardiac and cerebrovascular events, Non-ST elevation myocardial infarction, SYNTAX scores, Total bilirubin

## Abstract

**Background:**

The potential prognostic role of total bilirubin (TBIL) in patients with new-onset non-ST elevation myocardial infarction (NSTEMI) is not fully understood. This study aims to evaluate the potential predictive value of TBIL for long-term prognosis in patients with new-onset NSTEMI.

**Methods:**

Patients with new-onset NSTEMI that underwent emergency coronary angiography in our department from June 2015 to March 2020 were included. Baseline TBIL was measured at admission. SYNTAX scores were used to indicate the severity of coronary lesions. The association between TBIL and SYNTAX scores was analyzed using multivariate logistic regression. The patients were followed for the incidence of major adverse cardiac and cerebrovascular events (MACCEs). The association between TBIL and MACCEs was analyzed using Kaplan–Meier survival methods.

**Results:**

In total 327 patients were included in this study. Patients were divided according to tertiles of TBIL (first tertile < 10.23 µmol/L, n = 109; second tertile 10.23–14.30 µmol/L, n = 109; and third tertile ≥ 14.30 µmol/L, n = 109). TBIL was independently associated with the severity of coronary lesions in patients with NSTEMI, with an adjusted odds ratio (OR) and 95% confidence interval (CI) for the third tertile and the second tertile compared with the first tertile of TBIL of 2.259 (1.197–4.263) and 2.167 (1.157–4.059), respectively (both *p* < 0.05). After a mean follow-up of 30.33 months, MACCE had occurred in 57 patients. TBIL was independently associated with the increased risk of MACCEs, with an adjusted hazard ratio (HR) and 95% CI for the third tertile and the second tertile compared with the first tertile of TBIL of 2.737 (1.161–6.450) and 3.272 (1.408–7.607), respectively (both *p* < 0.05).

**Conclusions:**

Higher myocardial infarction admission TBIL might independently predict poor prognosis in patients with NSTEMI.

## Introduction

The pathological features of acute coronary syndrome (ACS) involve inflammation and oxidative stress that have been associated with conventional risk factors for coronary artery disease (CAD), such as diabetes mellitus, smoking, and hypertension [1–3]. However, the evidence suggests that some individuals without the previous risk factors could develop ACS, which suggests that there are potential unknown risk factors for CAD in these patients [4–6]. Clinically, non-ST elevation myocardial infarction (NSTEMI) is a subtype of non-ST elevation ACS (NSTE-ACS), which usually is associated with a more severe clinical status and worse outcomes than patients with unstable angina (UA), the other subtype of NSTEACS [7]. Therefore, identification of the novel risk factors that might predict the prognosis in patients with NSTEMI is of important clinical significance in current cardiovascular practice.

Previous studies have confirmed that bilirubin, which is a product of heme metabolism, could potentially exert endogenous anti-oxidative and anti-inflammatory efficacies at the physiological level [8]. Under pathological conditions, bilirubin could modulate the progression of atherosclerosis by the inhibition of the oxidative modification of low-density lipoprotein and proliferation of smooth muscle cells (SMC) [8]. However, elevated bilirubin post-myocardial infarction might reflect increased heme breakdown that includes increased red cell mass, heme oxygenase 1 enzyme (HO-1) expression, myoglobin breakdown, and decreased hepatic bilirubin glucuronidation, or both caused by reduced hepatic blood flow following myocardial infarction [9]. Therefore, previous clinical studies have suggested that higher serum levels of total, direct, and indirect bilirubin might be associated with an increased risk of the combined outcomes of major adverse cardiac and cerebrovascular events (MACCEs) in patients with ACS, which include all-cause death, myocardial infarction, and stroke [10, 11]. However, some of the previous studies have indicated that total bilirubin (TBIL) might confer better prognostic efficacy than direct or indirect bilirubin in ACS patients [12, 13], other studies that evaluated the predictive role of serum TBIL in ACS patients based on the subtype of ACS showed inconsistent results [11, 13–15]. Some of the studies did not support that serum TBIL was associated with an increased risk of MACCEs in ACS patients [14–16]. In addition, the sample sizes of previous studies were limited, and patients with previously diagnosed CAD were included, which might affect the results of the studies. Because of the important role of inflammation in the pathogenesis of NSTEMI, as well as the potential role of bilirubin as an endogenous anti-inflammatory factor, this study aims to systematically evaluate the potential associations between serum TBIL with severity and prognosis in patients with new-onset NSTEMI.

## Methods

### Patients and study design

**Fig. 1:**
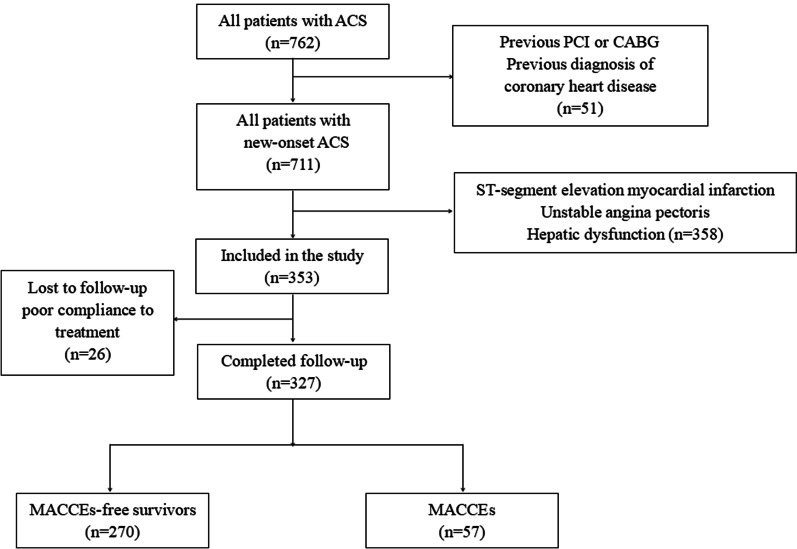
Flowchart of patient enrollment

Patients with new-onset NSTEMI and without previously known CAD that underwent urgent coronary angiography in the Xinjiang Medical University Affiliated Hospital of Traditional Chinese Medicine from June 2015 to March 2020 were included in this study. The diagnosis of NSTEMI was based on the criteria in previous guidelines [17]. New-onset NSTEMI was defined as a first episode of new-onset NSTEMI without previously known CAD. Patients with any of the following clinical conditions were excluded: (1) hepatic or renal dysfunction that might affect serum TBIL; (2) diagnosis of ST-segment elevation myocardial infarction (STEMI), unstable angina pectoris, or with previous revascularization therapy, which included percutaneous coronary intervention (PCI) and coronary artery bypass grafting (CABG); (3) new-onset NSTEMI with a previous diagnosis of CAD; (4) patients that had pacemaker-implantation, malignant tumors, or severe infection; (5) patients with previously diagnosed systemic inflammatory disease, a history of alcohol consumption, hemolysis, blood transfusion, viral infections of the liver, or with poor compliance to treatment; and (6) patients who were at risk of hepatotoxicity induced by medications, such as the use of statins or amidarone. The study was approved by the Ethics Committee of The Xinjiang Medical University Affiliated Hospital of Traditional Chinese Medicine (No. 2022XE0117). Because this was a retrospective observational study, the ethics committee of the Xinjiang Medical University Affiliated Hospital of Traditional Chinese Medicine waived the requirement for informed consent from eligible patients. All methods were performed in accordance with the relevant guidelines and regulations. The flow chart of participant enrollment is shown in Fig. 1.

### Blood sampling

Peripheral venous blood samples were drawn immediately before urgent coronary angiography for each of the patients and sent to the Department of Clinical Laboratory of the Xinjiang Medical University Affiliated Hospital of Traditional Chinese Medicine for further analysis. Parameters of blood cell count, biochemical parameters for lipids and glucose metabolism, hepatic and renal function, serum uric acid, serum creatine phosphokinase-MB (CK-MB), and troponin T were measured.

### Coronary angiography and SYNTAX score

After admission, all patients underwent emergency coronary angiography using a standard protocol that was carried out by experienced cardiologists. Emergency coronary angiography was defined as coronary angiography within 12 h of angina onset. The SYNTAX score was used as the indicator for the severity of the coronary lesions, which was calculated by two experienced cardiologists independently according to the online tool of the score. If disagreement occurred, they were resolved by consensus with the third investigator. If indications for PCI were detected, the modality of PCI was determined by a group of experienced attending physicians based on coronary anatomy and the clinical status of the individual patients. After PCI, patients continued with optimized medical treatments and were followed-up at clinics regularly after discharge.

### Follow-up

Patients were discharged and followed-up by telephone interview or clinic visits. All events were carefully monitored by an independent panel of clinical physicians. The primary outcome of this study was the incidence of a combined outcome for MACCEs, which included cardiac mortality, myocardial infarction, stent thrombosis, stroke and revascularization. The secondary outcome of this study was all-cause mortality.

### Statistical analysis

Patients were grouped based on the tertiles of the serum TBIL (first tertile < 10.23 µmol/L, second tertile 10.23–14.30 µmol/L, and third tertile ≥ 14.30 µmol/L, with 109 patients in each tertile) or tertiles of the SYNTAX score at baseline. Continuous variables were summarized as mean and standard deviation if normally distributed; otherwise, medians and interquartile ranges (IQRs) were used. Categorical variables were expressed as percentages. Comparisons with means between multiple groups were performed using ANOVA, and for the nonnormally distributed variables, Mann–Whitney U test or Kruskal–Wallis variance analysis was applied. For the categorical variables, a Chi-squared (χ^2^) test was employed. Multiple logistic regression analysis was performed to identify the independent factors that were associated with the severity of coronary lesions, as evidenced by the SYNTAX score. The potential predictive efficacy of serum TBIL at baseline for prognosis in NSTEMI patients was analyzed using the Kaplan–Meier survival method. Univariate analysis was performed first, and then the significant variables were included in the multivariate Cox regression analysis. A *p* value < 0.05 indicated a statistically significant difference. SPSS 23 was used for the statistical analysis.

## Results

### Characteristics of the included patients

In total, 327 patients with new-onset NSTEMI were retrospectively included in this study. The baseline characteristics for all the patients included in this study based on the tertiles of TBIL (first tertile < 10.23 µmol/L, n = 109; second tertile 10.23–14.30 µmol/L, n = 109; and third tertile ≥ 14.30 µmol/L, n = 109) are presented in Table 1. The results showed that patients with higher TBIL levels were probable to be male and smokers, with higher apolipoprotein A1, (Apo-AI), increased high-density lipoprotein cholesterol, and a higher SYNTAX score (all *p* < 0.05). The baseline characteristics for all the patients in this study were based on tertiles of the SYNTAX score as given in Table 2. Age, the prevalence of diabetes mellitus, smoking status, left ventricle ejection fraction (LVEF), TBIL, and TBIL tertiles group were significantly different between the patients based on the tertiles of SYNTAX score (all *p* < 0.05).

**Table 1:** Baseline characteristics of included patients with NSTEMI according to TBIL tertiles

Clinical characteristics	First tertile < 10.23 µmol/L (n = 109)	Second tertile 10.23–14.30 µmol/L (n = 109)	Third tertile ≥ 14.30 µmol/L (n = 109)	*t/Z/χ*^2^	*p *value
Male (%)	65 (59.6)	70 (64.2)	82 (75.2)	6.274	**0.043**
Age (years)	60.88 ± 11.50	60.60 ± 8.96	59.61 ± 12.28	0.397	0.672
Hypertension (%)	44 (40.4)	58 (53.2)	53 (48.6)	3.704	0.157
Diabetes mellitus (%)	30 (27.5)	35 (32.1)	35 (32.1)	0.720	0.698
Current smoking (%)	41 (37.6)	56 (51.4)	59 (54.1)	6.840	**0.033**
SBP (mm/Hg)	126.09 ± 23.38	129.06 ± 18.08	126.04 ± 17.09	0.836	0.434
DBP (mm/Hg)	74.94 ± 12.44	76.13 ± 11.02	76.33 ± 10.53	0.478	0.621
BMI (kg/m^2^)	25.09 ± 3.26	25.43 ± 4.89	25.83 ± 3.56	0.747	0.475
HDL-C (mmol/L)	0.91 (0.77, 1.11)	0.97 (0.82, 1.21)	0.98 (0.86, 1.17)	6.090	**0.048**
LDL-C (mmol/L)	2.73 ± 0.93	2.82 ± 0.94	2.74 ± 1.28	0.222	0.801
TC (mmol/L)	4.22 ± 1.16	4.31 ± 1.18	4.26 ± 1.52	0.147	0.863
TG (mmol/L)	1.57 (1.12, 2.43)	1.56 (1.15, 2.56)	1.36 (0.98, 2.40)	3.595	0.166
Apo-A1 (g/L)	1.07 (0.95, 1.23)	1.20 (1.01, 1.38)	1.13 (0.97, 1.38)	8.441	**0.015**
Apo-B (g/L)	0.81 (0.64, 1.01)	0.87 (0.70, 1.09)	0.77 (0.62, 1.09)	2.735	0.255
Creatinine (mmol/L)	70.00 (61.23, 81.00)	71.00 (59.16, 79.90)	75.00 (63.38, 88.29)	4.607	0.100
BUN (mmol/L)	5.62 ± 3.10	5.16 ± 1.60	5.22 ± 1.72	1.383	0.252
Uric acid (mmol/L)	316.83 ± 84.14	296.37 ± 90.88	321.66 ± 102.25	2.284	0.104
CK-MB (U/L)	37.99 (15.71, 65.07)	32.72 (17.08, 79.90)	53.41 (21.25, 85.47)	4.229	0.121
Troponin T (μg/L)	0.40 (0.13, 1.15)	0.54 (0.18, 1.25)	0.63 (0.22, 1.33)	5.125	0.077
LVEF (%)	60.83 ± 6.00	60.36 ± 5.64	60.83 ± 6.48	0.144	0.866
LVEDD (mm)	49.96 ± 4.41	50.07 ± 3.42	49.24 ± 4.56	0.867	0.422
Killip class (%)				4.414	0.621
I	4 (3.7)	2 (1.8)	3 (2.8)		
II	84 (77.1)	94 (86.2)	93 (85.3)		
III	16 (14.7)	10 (9.2)	11 (10.1)		
IV	5 (4.6)	3 (2.8)	2 (1.8)		
GRACE Score	129.55 ± 42.20	130.65 ± 31.84	133.44 ± 32.88	0.237	0.790
CRUSADE Score	24.00 (14.00, 37.00)	23.00 (15.00, 34.50)	23.00 (13.00, 33.00)	0.218	0.897
SYNTAX Score	11.50 (6.00, 23.50)	16.00 (6.50, 23.50)	18.00 (9.25, 25.50)	6.953	**0.031**
Coronary lesions					
UPLMT (%)	11 (10.1)	13 (11.9)	19 (17.4)	2.785	0.248
LAD (%)	94 (86.2)	92 (84.4)	90 (82.6)	0.558	0.757
LCX (%)	79 (72.5)	985 (78.0)	80 (73.4)	1.001	0.606
RCA (%)	84 (77.1)	78 (71.6)	69 (63.3)	5.043	0.080
PCI (%)	76 (69.7)	81 (74.3)	80 (73.4)	0.644	0.725
Medications after discharge					
Aspirin (%)	101 (92.7)	107 (98.2)	101 (92.7)	4.233	0.120
Clopidogrel (%)	93 (85.3)	101 (92.7)	98 (89.9)	3.136	0.209
β-Blockers (%)	99 (90.8)	104 (95.4)	99 (90.8)	2.166	0.339
ACEI/ARB (%)	80 (73.4)	90 (82.6)	82 (75.2)	2.907	0.234
CCB (%)	81 (74.3)	84 (77.1)	74 (67.9)	2.457	0.293

Values of *p* < 0.05 are indicated in bold

ACEI, angiotensin-converting enzyme inhibitor; Apo-AI, apolipoprotein A1; Apo-B, apolipoprotein B; ARB, angiotensin II receptor blocker; BMI, body mass index; BUN, blood urea nitrogen; CCB, calcium channel blocker; Cr, creatinine; CK-MB, creatine kinase-MB; DBP, diastolic blood pressure; HDL-C, high-density lipoprotein cholesterol; LAD, left anterior descending artery; LCX, left circumflex artery; LDL-C, low-density lipoprotein cholesterol; LVEDD, left ventricular end-diastolic dimension; LVEF, left ventricle ejection fraction; PCI, percutaneous coronary intervention; RCA, right coronary artery; SBP, systolic blood pressure; TC, total cholesterol; TG, triglyceride; UPLMT, unprotected left main trunk

**Table 2:** Baseline characteristics of included patients according to the SYNTAX score tertiles

Clinical characteristics	First tertile (< 10.0, n = 107)	Second tertile (10–22, n = 111)	Third tertile (≥ 23, n = 109)	*F/Z/χ*^2^	*p *value
Male (%)	75 (70.1)	84 (75.7)	79 (72.5)	0.865	0.649
Age (years)	58.97 ± 10.15	59.50 ± 11.04	62.61 ± 11.45	3.524	**0.031**
Hypertension (%)	47 (43.9)	55 (49.5)	58 (53.2)	1.889	0.389
Diabetes mellitus (%)	23 (21.5)	36 (32.4)	41 (37.6)	6.880	**0.032**
Current smoking (%)	41 (38.3)	54 (48.6)	61 (56.0)	6.799	**0.033**
SBP (mm/Hg)	126.68 ± 22.06	127.35 ± 18.28	127.13 ± 18.88	0.032	0.969
DBP (mm/Hg)	77.05 ± 11.58	75.87 ± 11.82	74.58 ± 10.57	1.278	0.280
BMI (kg/m^2^)	25.67 ± 3.32	25.61 ± 3.73	25.09 ± 4.65	0.540	0.583
HDL-C (mmol/L)	0.97 (0.83, 1.24)	0.96 (0.80, 1.14)	0.95 (0.79, 1.16)	1.291	0.524
LDL-C (mmol/L)	2.71 ± 0.86	2.72 ± 1.10	2.85 ± 1.17	0.539	0.584
TC (mmol/L)	4.12 ± 1.05	4.27 ± 1.32	4.4 ± 1.45	1.238	0.291
TG (mmol/L)	1.47 (1.04, 2.35)	1.49 (1.08, 2.44)	1.56 (1.12, 2.58)	1.225	0.542
Apo-A1 (g/L)	1.15 (1.01, 1.37)	1.08 (0.99, 1.28)	1.15 (0.95, 1.34)	2.506	0.286
Apo-B (g/L)	0.80 (0.68, 1.05)	0.79 (0.64, 1.02)	0.90 (0.63, 1.10)	2.521	0.284
Creatinine (mmol/L)	70.56 (60.52, 82.22)	71.00 (63.00, 84.00)	72.14 (62.18, 82.00)	0.404	0.817
BUN (mmol/L)	5.27 ± 2.35	5.01 ± 1.63	5.71 ± 2.61	2.797	0.062
Uric acid (mmol/L)	307.07 ± 98.73	317.15 ± 82.57	310.31 ± 98.09	0.330	0.719
CK-MB (U/L)	39.41 (16.03, 70.00)	35.35 (16.25, 74.00)	44.19 (21.15, 89.36)	2.364	0.307
Troponin T (µg/L)	0.49 (0.18, 1.30)	0.56 (0.16, 1.26)	0.45 (0.16, 1.16)	0.075	0.963
LVEF (%)	61.28 ± 5.31	61.55 ± 5.56	58.97 ± 7.04	3.895	**0.022**
LVEDD (mm)	49.24 ± 4.41	49.33 ± 3.47	50.76 ± 4.49	2.970	0.053
Killip class (%)				7.097	0.312
I	4 (3.7)	3 (2.7)	2 (1.8)		
II	93 (86.9)	93 (83.8)	85 (78.0)		
III	9 (8.4)	12 (0.8)	16 (14.7)		
IV	1 (0.9)	3 (2.7)	6 (5.5)		
GRACE Score	132.39 ± 41.11	125.67 ± 28.98	135.26 ± 34.75	1.461	0.234
CRUSADE Score	22.00 (12.50, 35.00)	23.00 (14.00, 31.00)	25.00 (15.25, 36.00)	1.185	0.553
TBIL (mmol/L)	10.50 (8.62, 15.59)	13.00 (10.20, 16.00)	13.18 (9.66, 16.88)	8.283	**0.016**
TBIL tertiles				14.404	**0.006**
First tertile	50 (46.7)	30 (27.0)	29 (26.6)		
Second tertile	29 (27.1)	44 (39.6)	36 (33.0)		
Third tertile	28 (26.2)	37 (33.3)	44 (40.4)		

Values of *p* < 0.05 are indicated in bold

Apo-AI, apolipoprotein A1; Apo-B, apolipoprotein B; BMI, body mass index; BUN, blood urea nitrogen; DBP, diastolic blood pressure; CK-MB, creatine kinase-MB; HDL-C, high-density lipoprotein cholesterol; Lp(a), lipoprotein (a); LAD, left anterior descending artery; LDL-C, low-density lipoprotein cholesterol; LVEDD, left ventricular end-diastolic dimension; LVEF, left ventricle ejection fraction; SBP, systolic blood pressure; TC, total cholesterol; TG, triglyceride; TBIL, total bilirubin

### Potential association between TBIL and severity of coronary lesions

The results of multivariate logistic analyses showed that a higher TBIL was independently associated with the severity of coronary lesions based on the SYNTAX score, with an adjusted odds ratio (OR) and 95% confidence interval (CI) for the third tertile and the second tertile compared with the first tertile of TBIL of 2.259 (1.197– 4.263) and 2.167 (1.157–4.059), respectively as given in Table 3 (both *p* < 0.05) In addition, other factors that include diabetes (OR 1.954, *p* = 0.016), smoker (OR 1.829, *p* = 0.023), and LVEF (OR 0.954, *p* = 0.032; Table 3) were associated with the severity of coronary lesions in patients with new-onset NSTEMI.

**Table 3:** Independent predictors of coronary lesion severity as detected by SYNTAX score

Variables	*B*	*SE*	*Wald*	*p *value	*OR*	*95% CI*	
						Lower limit	Upper limit
Age	0.011	0.013	0.706	0.401	1.011	0.986	1.036
LVEF (%)	-0.047	0.022	4.586	**0.032**	0.954	0.914	0.996
Diabetes mellitus	0.670	0.279	5.777	**0.016**	1.954	1.132	3.377
Current smoking	0.604	0.266	5.132	**0.023**	1.829	1.084	3.083
TBIL tertiles							
2nd tertile versus 1st tertile	0.815	0.324	6.322	**0.012**	2.259	1.197	4.263
3rd tertile versus 1st tertile	0.773	0.320	5.834	**0.016**	2.167	1.157	4.059

Values of *p* < 0.05 are indicated in bold

OR, odds ratio; CI, confidence interval; LVEF, left ventricular ejection fraction

### Incidence of MACCEs and all-cause mortality according to the TBIL

The incidences of primary and secondary clinical outcomes during a mean follow-up of 30.33 months for the patients in this study with new-onset NSTEMI, based on the tertiles of TBIL at baseline are given in Table 4. During follow-up, 57 patients experienced MACCEs. The results showed that the incidence of MACCEs increased in patients based on the tertiles of serum levels of TBIL (*p* = 0.001). However, the incidence of all-cause mortality was not statistically different between patients with new-onset NSTEMI, based on the tertiles of TBIL at baseline (*p* = 0.177).

**Table 4:** Incidence of adverse outcomes in new-onset NSTEMI patients according to the TBIL tertiles

Variables	1st tertile < 10.23 umol/L (n = 109)	2nd tertile 10.23–14.30 μmol/L (n = 109)	3rd tertile ≥ 14.30 μmol/L (n = 109)	*χ*^2^	*p *value
MACCEs, n (%)	7 (6.4)	23 (21.1)	27 (24.8)	14.278	**0.001**
Sudden cardiac death, n (%)	7 (6.4)	5 (4.6)	8 (7.3)	0.746	0.689
Recurrent MI, n (%)	3 (2.8)	6 (5.5)	7 (6.4)	1.709	0.426
Revascularization, n (%)	5 (4.6)	14 (12.8)	18 (16.5)	9.041	**0.011**
Stroke, n (%)	0 (0.0)	4 (3.7)	4 (3.7)	6.587	**0.037**
Stent thrombosis, n (%)	4 (3.7)	5 (4.6)	4 (3.7)	0.157	0.925
All-cause mortality, n (%)	4 (3.7)	8 (7.3)	11 (10.1)	3.461	0.177

Values of *p* < 0.05 are indicated in bold

MI, myocardial infarction; MACCEs, major adverse cardiac and cerebrovascular events

### Predictors of clinical outcomes

**Fig. 2:**
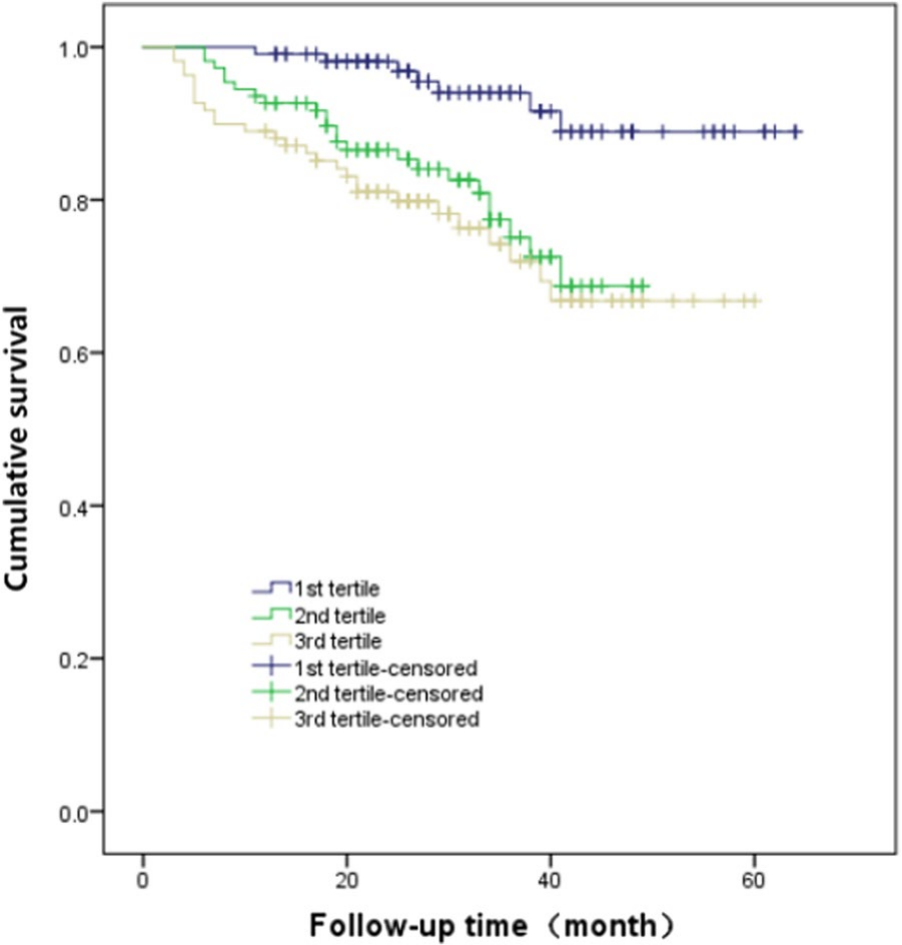
Cumulative incidence of MACCE in patients with NSTEMI according to TBIL tertiles

**Fig. 3:**
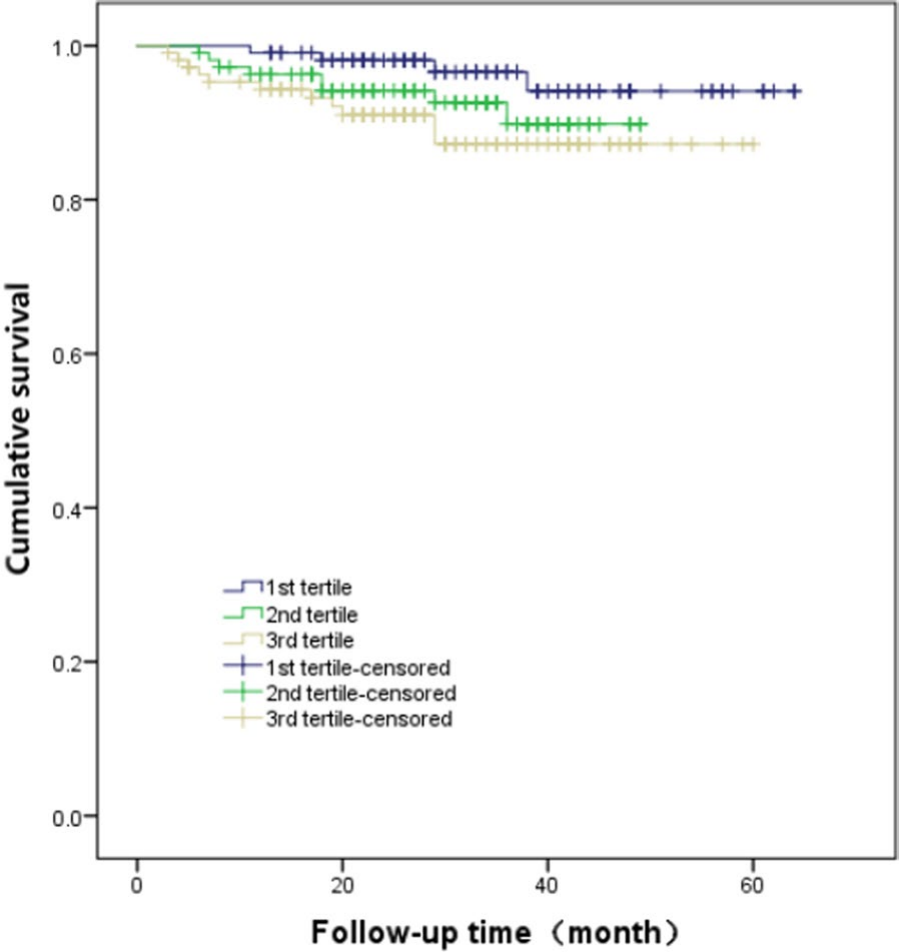
Cumulative incidence of all-cause mortality in patients with NSTEMI according to TBIL tertiles

Kaplan–Meier analysis demonstrated that the incidence of MACCEs was significantly different among patients with new-onset NSTEMI based on the tertiles of TBIL (χ^2^ = 15.243, *p* < 0.001) as shown in Fig. 2 and the incidence of all-cause mortality was not significantly different among patients based on the tertiles of TBIL (χ^2^ = 4.430, *p* = 0.109) as shown in Fig. 3. The Results of univariate Cox regression analysis indicated that gender (female), hypertension, diabetes, increased troponin T, unprotected left main trunk coronary artery lesions, higher TBIL tertile, and higher SYNTAX score tertile were potential predictors of MACCEs, as given in Table 5 (*p* values all < 0.05). Subsequent multivariate analysis showed that TBIL was independently associated with an increased risk of MACCEs, with adjusted hazard ratio (HR) and 95% CI for the third tertile and the second tertile compared with the first tertile of TBIL of 2.737 (1.161–6.450) and 3.272 (1.408–7.607), respectively (both p < 0.05). Other independent risk factors for the increased incidence of MACCEs in patients with new-onset NSTEMI included diabetes (HR 1.800, 95% CI 1.041–3.113, p = 0.035), UPLMCA (HR 2.042, 95% CI 1.063–3.923, *p* = 0.032), increased troponin T (HR 1.172, 95% CI 1.007–1.365, p = 0.040), and increased SYNTAX scores (third tertile versus first tertile, HR 3.165, 95% CI 1.280–7.827, p = 0.013); and second tertile versus first tertile (HR 2.767, 95% CI 1.097–6.980, p = 0.031) as given in Table 5.

**Table 5:** Predictors for the incidence of MACCEs in patients with new-onset NSTEMI

Variables	Univariate analysis			Multivariate analysis		
	*HR*	*95% HR*	*p *value	*HR*	*95% CI*	*p *value
Female, n (%)	0.533	0.287–0.991	**0.047**	0.598	0.319–1.121	0.109
Age (years)	1.011	0.987–1.035	0.387			
Hypertension, n (%)	1.774	1.044–3.012	**0.034**	1.296	0.742–2.261	0.362
Diabetes mellitus, n (%)	2.130	1.259–3.602	**0.005**	1.800	1.041–3.113	0.035
Current Smoking, n (%)	1.211	0.720–2.036	0.470			
SBP (mmHg)	1.001	0.988–1.014	0.860			
DBP (mmHg)	0.992	0.969–1.015	0.480			
BMI (kg/m^2^)	0.958	0.892–1.029	0.237			
HDL-C (mmol/L)	0.517	0.190–1.405	0.196			
LDL-C (mmol/L)	0.891	0.685–1.158	0.388			
TC (mmol/L)	0.956	0.777–1.175	0.667			
TG (mmol/l)	1.001	0.937–1.068	0.996			
ApoA1 (g/L)	0.736	0.311–1.741	0.485			
ApoB (g/L)	0.676	0.284–1.610	0.377			
Creatinine (mmol/L)	0.995	0.984–1.006	0.390			
BUN (mmol/l)	0.943	0.818–1.086	0.412			
Uric acid (mmol/L)	0.998	0.996–1.001	0.299			
CK-MB (U/L)	1.001	0.998–1.002	0.674			
Troponin T (μg/L)	1.232	1.057–1.435	**0.007**	1.172	1.007–1.365	0.040
TBIL tertiles						
2nd tertile vs 1st tertile	3.653	1.566–8.518	**0.003**	2.737	1.161–6.450	**0.021**
3rd tertile vs 1st tertile	4.555	1.983–10.465	**< 0.001**	3.272	1.408–7.607	**0.006**
LVEF (%)	0.986	0.942–1.032	0.542			
LVEDD (mm)	0.990	0.921–1.066	0.797			
Killip class	0.953	0.570–1.595	0.855			
GRACE Score	0.997	0.989–1.006	0.551			
CRUSADE Score	0.996	0.979–1.013	0.635			
SYNTAX tertiles						
2nd tertile vs 1st tertile	3.40	1.366–8.469	**0.009**	2.767	1.097–6.980	**0.031**
3rd tertile vs 1st tertile	5.214	2.175–12.498	**< 0.001**	3.165	1.280–7.827	**0.013**
UPLMT, n (%)	2.932	1.641–5.240	**< 0.001**	2.042	1.063–3.923	**0.032**
PCI, n (%)	1.329	0.726–2.435	0.357			
Medications after discharge						
Aspirin, n (%)	3.097	0.429–22.380	0.263			
Clopidogrel, n (%)	2.820	0.687–11.586	0.150			
Statins, n (%)	2.298	0.561–9.423	0.248			
β-Blockers, n (%)	1.330	0.671–2.633	0.414			
ACEI/ARB, n (%)	1.119	0.612–2.045	0.715			
CCB, n (%)	1.111	0.561–2.202	0.762			

Values of *p* < 0.05 are indicated in bold. All abbreviations are presented in Table 1

## Discussion

In this retrospective cohort study that included patients with new-onset NSTEMI, a higher TBIL at baseline was independently associated with the severity of coronary lesions as shown by the higher SYNTAX score. In addition, with a mean follow-up of 30.33 months, higher serum TBIL at baseline was an independent predictor for an increased incidence of MACCEs in patients with new-onset NSTEMI. Because of the convenience and cost-effectiveness of measuring myocardial infarction admission TBIL in clinical practice, these results suggested that serum TBIL might be an inexpensive predictor for poor prognosis in patients with new-onset NSTEMI.

The risk stratification for patients with new-onset NSTEMI needs to be improved, in particular, for the identification of potential prognostic factors for these patients [7]. Although previous studies have suggested a potential role of TBIL as a prognostic factor in CAD, the results of these studies might be different based on the subtype of CAD. A previous study that included 7,685 healthy individuals with a mean follow-up of 11.5 years showed that higher TBIL might be a risk factor for the increased incidence of ischemic heart disease [18]. A retrospective study that included 3,013 patients with angiographically obstructive CAD suggested a positive and independent correlation between baseline levels of TBIL and short-term mortality of acute myocardial infarction patients, and the negative correlation between baseline levels of TBIL and long-term mortality in stable CAD or UA pectoris patients was confirmed in a cohort study with a follow-up of 1 year [19]. In addition, high serum TBIL levels have been independently and significantly correlated with the burden of coronary atherosclerosis in patients with STEMI, and no significant association between high serum TBIL levels and poor long-term prognosis was found in these studies [15, 20].

In this retrospective cohort study, a significant association was found between myocardial infarction admission TBIL and the severity of coronary lesions. The results of our study are consistent with previous results, which demonstrated that LVEF was associated with the severity of coronary artery lesions in patients with CAD [21, 22]. In addition, compared with the known risk factors, which include LVEF, diabetes, and smoking, TBIL was one of the strongest factors that was correlated with the severity of coronary angiographic findings. In addition, TBIL acted as an effective and inexpensive predictor in new-onset NSTEMI, with higher TBIL admissions relative to a three3-fold increase in the risk of MACCEs after it was corrected for established predictive factors, such as troponin and SYNTAX score. Of note, TBIL might be a potential protective factor for coronary lesions based on the potential endogenous anti-oxidative and anti-inflammatory characteristics of bilirubin. In addition, it needs to be emphasized that the previous hypothesis was that myocardial infarction admission TBIL was elevated shortly after myocardial infarction because of the acute response to impaired liver function [23]. Besides, previous studies demonstrated that the TBIL changed dramatically as regulated by HO-1 and the maximal levels of bilirubin were usually observed during an acute myocardial infarction event [24, 25]. Moreover, STEMI patients with high bilirubin levels were shown to have a higher incidence of adverse outcomes and mortality during hospitalization [26], which suggested a role for increased TBIL as a predictor for poor prognosis in STEMI patients. This study, by strictly excluding patients with a previous diagnosis of CAD and other concurrent comorbidities that might affect the TBIL level, showed that higher TBIL at baseline was independently associated with a higher risk of MACCEs in new-onset NSTEMI patients. These findings support the incorporation of baseline TBIL levels for risk stratification of patients with new-onset NSTEMI. Our data indicated that where present, attention should be given to atypical chest pains patients with unexplained elevated TBIL, regardless of other risk factors. These patients should undergo comprehensive cardiovascular evaluation and intervention. In addition, appropriate preventative programs should be tailored to new-onset NSTEMI patients with increased TBIL.

The pathophysiological mechanisms that underly the association between bilirubin and poor prognosis in patients with new-onset NSTEMI need to be determined. Based on previous studies, it could be hypothesized that acute myocardial ischemia might induce an immediate increase in the levels of various inflammatory cytokines and reduced hepatic blood flow, which might exceed the protective antioxidant effect of bilirubin in vivo [23]. In addition, the another inferred that there was a compensatory increase of TBIL by dramatically up-regulated HO-1 activity under stress to exert anti-inflammatory and anti-oxidative effects in new-onset NSTEMI patients [24, 25]. Our data is consistent with previous findings that patients with increased serum bilirubin levels had increased cardiac troponin I release that was correlated with myocardial infarction size and the severity of coronary atherosclerotic burden [27]. Therefore, high TBIL levels might have a protective anti-oxidative effect on the cardiovascular system in stable CAD and healthy population. In addition, it has been suggested that long-term therapy with statins or aspirin might be associated with increased TBIL levels [28, 29]. However, this appears not to be the main influencing factors in this study, because only new-onset NSTEMI patients without a previous diagnosis of CAD were included.

### Study limitations

Several limitations of this study are noted. First, this was a retrospective observational study with limited sample size, and the findings should be validated in large-scale prospective cohort studies. In addition, the serum TBIL was only measured once at admission, and whether dynamic changes in serum TBIL during hospitalization had a more significant impact on the prognosis of these patients is unknown. In addition, this was an observational study, and a causative association between increased serum TBIL and poor prognosis in these patients could not be derived based on the findings. Finally, the optimal cut-off value for the prognostic efficacy of TBIL is unknown, which deserves further investigation.

## Conclusions

The results of this study suggest that myocardial infarction admission TBIL might be an inexpensive predictor of poor prognosis in patients with new-onset NSTEMI. Because of the convenience and cost-effectiveness of measuring serum TBIL, the findings support the incorporation of the measurement of serum TBIL when risk stratification for patients with new-onset NSTEMI is performed.

## Data Availability

The datasets used and/or analyzed during this study are available from the corresponding author on reasonable request. Requests to access these datasets should be directed to Tongjian Zhu, whuzhutongjian@126.com.

## Abbreviations

ACEI: Angiotensin-converting enzyme inhibitor
Apo-AI: Apolipoprotein A1
Apo-B: Apolipoprotein B
ARB: Angiotensin II receptor blocker
BMI: Body mass index
BUN: Blood urea nitrogen
CCB: Calcium channel blocker
Cr: Creatinine
CK-MB: Creatine kinase-MB
DBP: Diastolic blood pressure
HDL-C: High-density lipoprotein cholesterol
LAD: Left anterior descending artery
LCX: Left circumflex artery
LDL-C: Low-density lipoprotein cholesterol
LVEDD: Left ventricular end-diastolic dimension
LVEF: Left ventricle ejection fraction
PCI: Percutaneous coronary intervention
RCA: Right coronary artery
SBP: Systolic blood pressure
TC: Total cholesterol
TG: Triglyceride
UPLMT: Unprotected left main trunk
